# Comprehensive approaches to design efficient gRNA for SDN1-CRISPR/Cas9 genome editing in wheat

**DOI:** 10.3389/fgeed.2025.1579165

**Published:** 2025-07-01

**Authors:** Tushadri Singh, H. M. Mamrutha, Rajender Singh, J. P. Jaiswal, Zeenat Wadhwa, Rakesh Kumar, Omvir Singh, O. P. Ahlawat, Ratan Tiwari

**Affiliations:** ^1^ ICAR-Indian Institute of Wheat and Barley Research, Karnal, India; ^2^ Department of Genetics and Plant Breeding, College of Agriculture, Govind Ballabh Pant University of Agriculture and Technology, Pantnagar, Uttarakhand, India

**Keywords:** gRNA, CRISPR/Cas9, wheat, genome editing, WheatCRISPR, SDN1

## Abstract

**Background:**

CRISPR/Cas9 technology has gained popularity due to its efficient, widely applicable, and relatively easy genome editing. Furthermore, the removal of regulation on site-directed nuclease1- (SDN1) and SDN2-developed products in many countries has made it a more revolutionary technology for adoption in crop improvement. Designing accurate guide RNA (gRNA) is the initial and most crucial step that decides the success of the editing. Although the gene editing technique is widely used in crops, a detailed and comprehensive method for designing efficient gRNA in wheat is still lacking. By virtue of wheat being a hexaploid crop and having a large genome size with repetitive DNA, a tailor-made strategy for designing the gRNA is crucial.

**Result:**

The manuscript explains the comprehensive strategies and methods for efficient gRNA designing by considering the physical and structural expression of the target gene in the genome and explains the on-target and off-target effects of gRNA for its precise editing through the CRISPR/Cas9-mediated SDN1 method of genome editing in wheat.

**Conclusion:**

The present manuscript is first of its kind to address the holistic approach, starting from efficient gene selection, gRNA designing, and post-gRNA designing issues like gRNA stability, binding efficiency, and functionality for SDN1-CRISPR/Cas9 genome editing in wheat. This manuscript will be a ready reference for wheat researchers designing effective gRNA for wheat improvement to meet future food demand.

## 1 Background

Genome editing technology, termed CRISPR (clustered, regularly interspersed, palindromic repeats)/Cas9 (CRISPR-associated endonuclease 9), derived from *Streptococcus pyogenes*’s bacterial adaptive immune system, has initiated a new chapter in genetic engineering (Gillette et al., 2002; Mojica et al., 2005; Doudna and Charpentier, 2014; Voytas and Gao, 2014). Recently, this technology has gained importance due to its relative ease of working and acceptance of edited plants in agriculture due to relaxed regulations in many countries (Abdul Aziz et al., 2022). The products developed through site-directed nuclease1 (SDN-1) and site-directed nuclease2 (SDN-2) are largely considered non-transgenics in many countries, such as India, the United States, Japan, Australia, and New Zealand. Many of their products are under field testing in different countries, and several have been commercially released (Abdul Aziz et al., 2022; Waltz, 2018; Menz et al., 2020; Sprink et al., 2022; Bruetschy, 2019). A third approach, site-directed nuclease3 (SDN-3), enables the **precise introduction of entire genes** and is similar to transgenics. Unlike SDN1 and SDN2, which mainly result in gene knockouts or small edits, SDN3 allows **gene replacement, trait addition, or complex genetic modifications**. Plants developed using SDN-3 techniques are subject to the same regulatory procedures as traditional genetically modified organisms (GMOs), necessitating rigorous risk assessments and approvals from regulatory bodies (Department of Biotechnology, 2022; Global Gene Editing Regulation Tracker, 2023). SDNs are faster and more targeted than other methods of conventional breeding as they are efficiently able to circumvent problems of different genome sizes, ploidy levels, repetitive regions, and heterozygosity while aiding breeders to access and target multiple genes at once (Hsu et al., 2013; Mahas and Mahfouz, 2018; Zaidi et al., 2020; Kawall, 2021; Doudna and Charpentier, 2014). Hence, this technology has a high potential for applications in improving consumer-preferred commercial traits and crop improvement to meet the future food demand of the growing population (Verma et al., 2023).

By minimizing genome engineering to a two-component system, genome editing has become easier than previous methods. CRISPR/Cas9 technology relies on two important components: first, a DNA-binding domain made of a single guide RNA (sgRNA) formed by fusing two small RNA molecules, namely, CRISPR RNA (crRNA) and an auxiliary trans-activating crRNA (tracrRNA), and second, a DNA-cleaving domain comprising a Cas9 endonuclease. Both components work together to guide the Cas9 to a specific DNA site to bring about cleavage of the target strand (Deltcheva et al., 2011; Barrangou et al., 2012; Gasiunas et al., 2012; Sander and J Keith, 2014; Hsu et al., 2013). Each crRNA unit contains a 20-nucleotide guide sequence complementary to a target site, designated as guide RNA (gRNA). It is gRNA that enables specificity (a variable part of gRNA designing) in every gene editing experiment by targeting the specific gene at a specific locus in the whole genome and bringing about the editing sought. Designing the correct, highly specific gRNA, which is unique for every gene, is the most crucial step on which the final success of the CRISPR/Cas9 editing depends. The gRNA sequence defines the region to be recognized by Cas9 for cleavage. An inappropriate gRNA leads to the production of sub-optimal, unintended, and ambiguous results that pose a bottleneck in the progress of editing the desired gene. Designing gRNA that is highly specific (high on-target activity) and possessing low off-target hits is thus a pre-requisite for editing the gene, and it depends on several factors, with the most important being the target crop.

Although CRISPR/Cas9 technology in diploid crops such as rice has met with considerable success, similar progress has not yet been achieved in wheat due to its complex allopolyploid genome (2n = 6x = 42) and huge genome size (17.1 Gb) compared to other crops. This makes it difficult to apply the general rules of gRNA designing to it directly. Coupled with this is the huge proportion of repetitive DNA sequences (more than 80% of the wheat genome) and the presence of multi-gene families, which make designing the gRNA still more complex in wheat (Garbus et al., 2015; Cram et al., 2019). The polyploidy nature of the crop increases the possibility of off-target mutations and decreases genome editing specificity (Kim et al., 2018). An *in silico* analysis revealed that the wheat A/D genome contains nearly 114,081,000/99,766,831 and 748,385/936,764 sequences specifically targetable by gRNAs in the form of 5′-GN(19–21)-GG-3′ in the wheat genome and complementary DNAs (cDNAs), respectively. It showed 21 and 22 targets per cDNA for the A and D genomes (considering 34,897 and 43,150 cDNAs for the A and D genomes, respectively) (Shan et al., 2013). Selecting a unique target site that has few genetically similar off-target sites throughout the genome can minimize off-target activity (O’Brien and Bailey, 2014). Therefore, a thorough understanding of the target gene and wheat genome for efficient gRNA designing is the foremost need of the hour.

Much literature explains the wheat gRNA designing methods in bits and pieces and can be difficult to comprehend (Smedley et al., 2021). Hence, this manuscript is the first to outline a consolidated, detailed method for effective gRNA designing in wheat. A novel approach was used, starting from intensive analysis of the target gene for SDN1 editing to address the intricacies of the wheat genome and optimizing specificity for minimizing off-target effects of designed gRNA. Also analyzed were the structural, physical, compositional, and free energy parameters of the gRNA using various bioinformatic tools to find the efficient gRNA with increased on-target effect for gene editing in wheat. Thus, this developed novel method acts as a ready reference for researchers to increase the precision and efficiency of SDN1-genome editing in wheat.

## 2 Materials and methods

### 2.1 Strategies for gRNA designing

**FIGURE 1:**
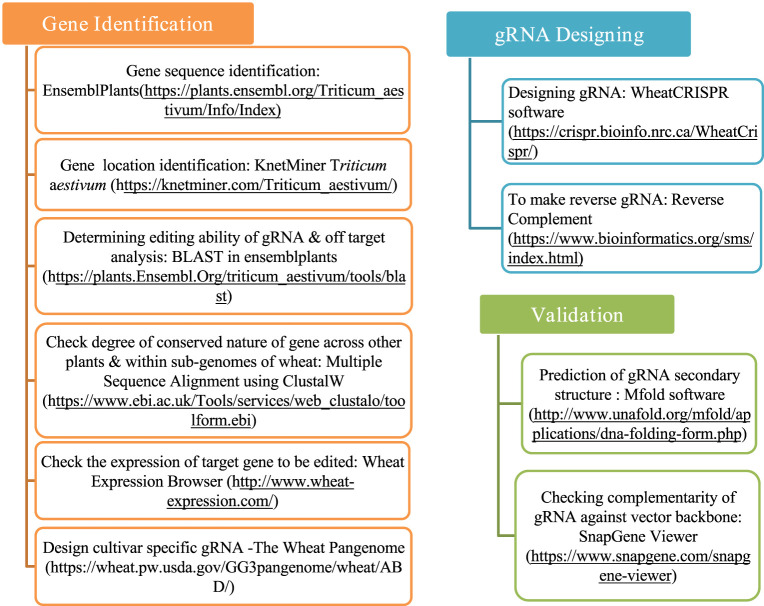
Comprehensive factors and databases to be considered for designing efficient gRNA for CRISPR/Cas9 genome editing in wheat.

The process of designing a gRNA for CRISPR/Cas9-SDN1 genome editing can be divided into three phases: gene verification, gRNA designing, and gRNA analysis. The potential target gene must be assessed in terms of having no pleiotropic effect, being qualitative in nature, negatively regulated, and should ideally have tissue/developmental stage-specific expression. Once the gene is identified, a gRNA is designed based on specific parameters. The designed gRNA is then validated by testing its potential secondary structure, Gibbs free energy, and its propensity to base pair within itself. Furthermore, its sequence similarity to the cloning binary vector to be used in the study should be checked. The various components that must be taken into account while designing an efficient gRNA are presented in Figure 1. Multiple software and databases can be utilized for validating gene verification, designing, and analyzing gRNA in wheat, as described below.

#### 2.1.1 Gene identification and verification

This step is critical to identify the gene, its nature, the chromosomal location, homologs, and the similarity across organisms and across the three sub-genomes of wheat. The most promising negative regulator gene for the SDN1-CRISPR/Cas9 study should be identified by an extensive review of literature about the same crop, about different crops through genome editing, or in knockout studies using RNA interference (RNAi)/targeting induced local lesions in genomes (TILLING). The target gene should not alter the final phenotype of the crop, except for the target trait and preferably should have tissue-specific expression rather than a pleiotropic effect in the crop. The Ensembl Plants database and KnetMiner *Triticum aestivum*, a bioinformatic tool, were used for gene sequence and gene location identification on wheat chromosomes (Yates et al., 2022; Hassani-Pak et al., 2021). To determine the editing ability of gRNAs in various sub-genomes and to identify off-targets, the Basic Local Alignment Search Tool (BLAST) was utilized. Clustal Omega software was used to assess the degree of similarity between the identified gene and genes present in other plant species and the three wheat sub-genomes. The Wheat PanGenome database (https://wheat.pw.usda.gov/GG3pangenome/wheat/ABD/) incorporates presence–absence variations, structural variants, and diverse allelic forms across wheat cultivars (Bayer et al., 2022; Hussain et al., 2022), and it supports precise cultivar-specific gRNA designing. By accessing genomic data across multiple cultivars, gRNAs targeting specific regions (either broadly conserved across wheat genomes or specific to a particular cultivar) can be designed (Figure 1).

#### 2.1.2 gRNA designing

WheatCRISPR software was used for gRNA designing (Cram et al., 2019). Subsequently, to get the complementary sequence of gRNA, reverse complement software was utilized. Required enzyme sites for cloning gRNA into the destination vector, if any, may be included before the synthesis of gRNA (Figure 1).

#### 2.1.3 gRNA analysis

The potential secondary structures of the designed gRNAs were predicted *in silico* using Mfold software (Zuker, 2003). SnapGene software was used before gRNA synthesis to check the complementary base pairing of gRNA, if any, against the destination vector backbone (GSL Biotech, 2020) (Figure 1).

## 3 Results and discussion

### 3.1 Target gene identification and verification

#### 3.1.1 Identifying commercially important traits and genes for SDN1-CRISPR/Cas9 editing

The process starts by identifying the commercially important traits that are most relevant to the objectives of the breeding program. These traits might include abiotic stress tolerance, resistance to pests and diseases, improved nutrient use efficiency, improved nutrition, or increased grain yield (Kumar et al., 2019). The literature review focuses on identifying genes that have been shown to regulate these traits, particularly those that act as negative regulators. These are the genes that, when suppressed or knocked out, lead to an enhancement of the desired trait. For example, the *TaGW2* gene in wheat, a well-known negative regulator of grain size, was recently edited using CRISPR/Cas9, leading to a significant increase in grain size and yield, demonstrating its potential as a target for genetic improvement (Hong et al., 2014).

Negative regulators are of particular interest in gene editing because their suppression or deletion often results in a positive effect on the trait of interest. The literature review aims to identify such genes across a wide range of studies, ensuring that the selected genes have been consistently associated with the trait (Table 1) (Hong et al., 2014; Zhang S. et al., 2021; Awan et al., 2024; Pearce et al., 2011; Jabłoński et al., 2020; Wang et al., 2014; Zhang et al., 2017; Zhang et al., 2016; Okada et al., 2019; Chen et al., 2022; Ouyang et al., 2016; BGRI, 2025; Raffan et al., 2021; Zhang et al., 2018; Zhang et al., 2019; Botticella et al., 2011; Ahmar and Gruszka, 2023; Tang et al., 2020; Wang et al., 2019).

**TABLE 1:** List of genes identified for SDN1-CRISPR/Cas9 genome editing in wheat.

Gene name	Trait targeted	Validated by	References
*TaGW2*	Grain size and yield improvement	CRISPR/Cas9	Hong et al. (2014)
*TaPinb*	Grain hardiness	CRISPR/Cas9	Zhang S. et al. (2021)
*TaD27*	Enhanced number of productive tillers	CRISPR/Cas9	Awan et al. (2024)
*TaRht-B1*	Dwarfism and improved yield	RNAi	Pearce et al. (2011)
*TaCKX1*	Cytokinin regulation and yield improvement	RNAi	Jabłoński et al. (2020)
*TaMLO*	Resistance to powdery mildew	CRISPR/Cas9	Wang et al. (2014)
*TaEDR1*	Enhanced resistance to powdery mildew	VIGS/RNAi	Zhang et al. (2017)
*TaGASR7*	Grain weight improvement	CRISPR/Cas9	Zhang et al. (2016)
*TaMS1*	Male sterility for hybrid seed production	CRISPR/Cas9	Okada et al. (2019)
*TaHRC*	Immune response and *Fusarium* head blight resistance	CRISPR/Cas9	Chen et al. (2022)
*TaPHO2-A1*	Improves phosphorus uptake and grain yield	Ion beam-induced deletion mutations	Ouyang et al. (2016)
*BT1*	Improved nitrogen-use efficiency	CRISPR/Cas9	BGRI (2025)
*TaASN2*	Low asparagine content in grain	CRISPR/Cas9	Raffan et al. (2021)
*DA1*	Increased yield and 1,000 kernel weight	CRISPR/Cas9	Zhang et al. (2018)
*TaCKX2-D1*	Increased grain number	CRISPR/Cas9	Zhang et al. (2019)
*Sbella*	Increased amylose content	EMS induced mutations	Botticella et al. (2011)
*ZnF-B*	Improved nitrogen-use efficiency by inhibiting brassinosteroid signaling	CRISPR/Cas9	Ahmar and Gruszka (2023)
*TaMS2*	Male sterility for hybrid seed production	CRISPR/Cas9	Tang et al. (2020)
*TaGW7*	Grain weight and size	CRISPR/Cas9	Wang et al. (2019)

#### 3.1.2 Prioritizing validated genes

Genes that have already been validated through experimental techniques such as gene knockout or silencing are prioritized. Validation techniques include RNAi, where the gene’s expression is reduced, and/or TILLING, where specific gene mutations are induced and their effects are studied (Mamrutha et al., 2023). For example, the cytokinin oxidase/dehydrogenase 1 gene (*TaCKX1*), which negatively regulates cytokinin levels in wheat, was targeted using RNAi, resulting in increased grain yield. This successful validation makes *TaCKX1* a high-priority candidate for CRISPR/Cas9-mediated genome editing (Jabłoński et al., 2020) (Table 1).

#### 3.1.3 Inter and cross-species comparison

The Wheat PanGenome database can be used to leverage the existing data effectively to design gRNA to identify conserved regions in target genes and examine homoeologous genes in sub-genomes. It also aids in cross-referencing with closely related cultivars. Using sequences from similar cultivars, sequence similarities can be inferred, providing a reasonable basis for gRNA designing (Montenegro et al., 2017). The Wheat Panache pangenome database covers 29 wheat varieties and includes a total of 2,490,453 genes. Within this vast gene pool, 78,319 genes are unique to specific varieties, showcasing the genetic diversity across different wheat cultivars. Additionally, the database identifies 9,789 core genes that are conserved across all varieties, representing essential functions shared within the wheat species. This extensive dataset allows examining common as well as unique genetic traits, aiding in the design of precise and cultivar-specific gRNAs. The availability of both core and unique genes enables targeted editing, helping to minimize off-target effects and enhancing the potential for trait-specific improvements in wheat. The Wheat Expression Browser (WEB) (http://www.wheat-expression.com/) was used to analyze the target gene expression in various tissues/organs (Borrill et al., 2016; Ramírez-González et al., 2018). It should be noted that, presently, wheat guide design tools refer to the Chinese Spring sequence databases. Therefore, there may be differences between the Chinese Spring sequence and the wheat cultivar being transformed and edited. To avoid such ambiguity, it is always preferred to sequence the target gene from the cultivar being transformed and align with the Chinese Spring sequences to check the differences in the gene sequences.

In addition to genes validated in wheat, promising genes for genome editing can also be identified in other related crops like rice, barley, and maize. These crops often share homologous genes with wheat, and finding genes from these species can provide valuable insights. For instance, the grain width2 (*OsGW2*) gene of rice functions in a similar manner to the grain width2 (*TaGW2*) gene of wheat (Hong et al., 2014) by controlling grain width and weight, and mutations in *OsGW2* led to increased grain size and enhanced yield (Achary and Reddy, 2021).

#### 3.1.4 Analyzing gene function and pathways

Another important aspect is to understand the biological pathways in which the candidate genes are involved. This involves reviewing studies on gene function, protein interactions, and metabolic pathways. For example, knocking out the asparagine synthetase2 (*TaASN2*) (Raffan et al., 2021) gene causes a reduction in free asparagine concentration in grain. By understanding how a gene interacts with other genes and proteins, researchers can predict the potential outcomes of editing that gene.

### 3.2 Gene expression verification

Understanding the expression pattern of the target gene is more critical for knock-out studies. The target gene expression can be assessed by checking the Wheat Expression Browser (WEB). The selected gene/s may either have a tissue-specific expression or expression in more than one tissue/organ, as discussed below.

#### 3.2.1 Gene having tissue-specific expression

**FIGURE 2:**
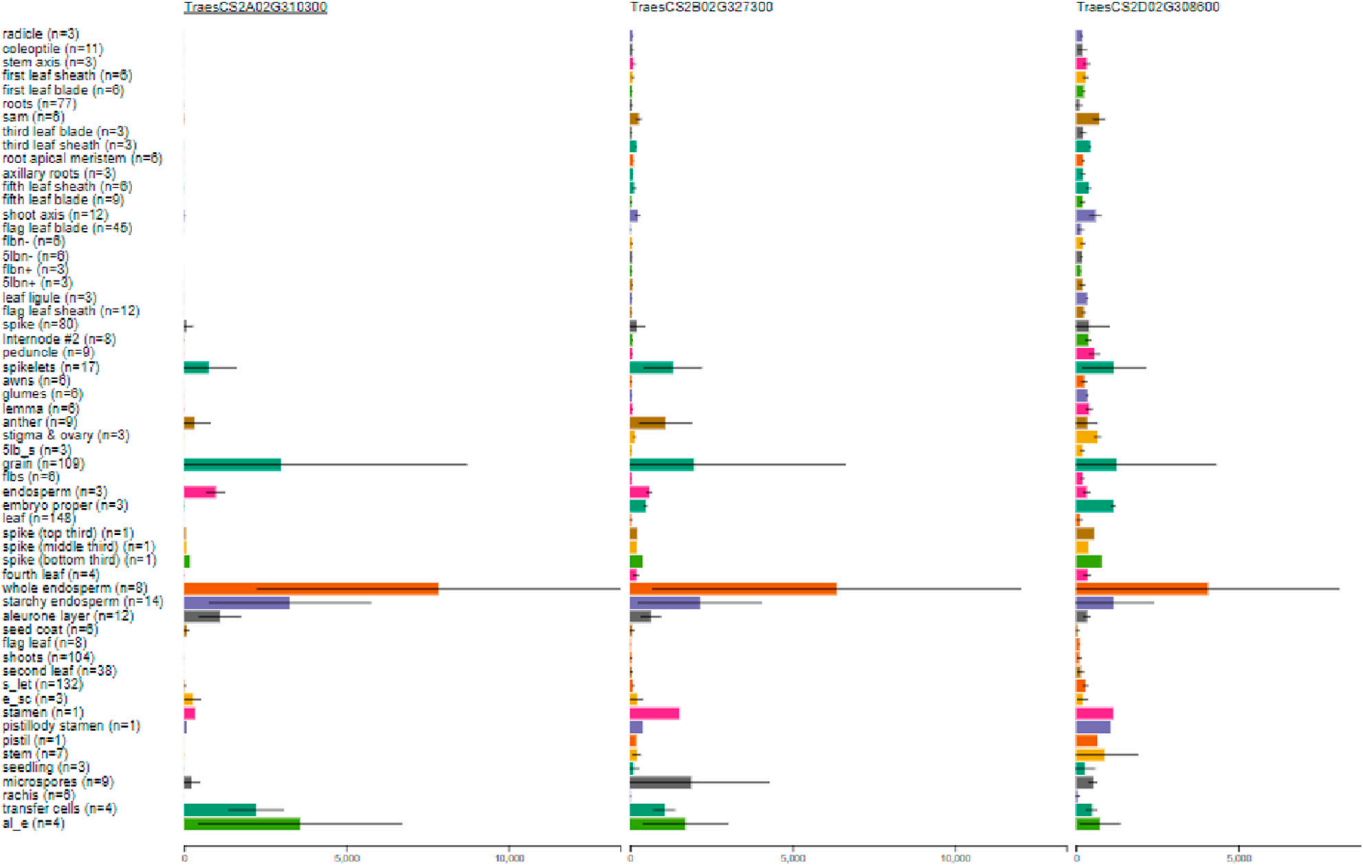
Tissue-specific expression (in transcripts per million) of the *SBEIIb* gene shown for the three homeologous gene IDs: TraesCS2A02G310300, TraesCS2B02G327300, and TraesCS2D02G308600 transcripts of A, B, and D sub-genomes, respectively. (Taken from http://www.wheat-expression.com/, where n is the number of studies that have reported the expression of the gene in a particular part of the plant).

Suppression of an isoform of starch-branching enzyme (SBE) II, that is*, SBEIIb* in wheat, is known to enhance the seed amylose content in rice in combination with other isoforms (Regina et al., 2015; Regina et al., 2006). The expression profile of *SBEIIb* was checked against the RefSeq1.1 nucleotide base using WEB. It was observed that the highest expression of the *SBEIIb* gene was present in the whole endosperm (Figure 2), showing a clear tissue-specific expression. Thus, this gene can be considered as a potential candidate gene for knock-out studies using CRISPR/Cas9.

#### 3.2.2 Gene having non-tissue-specific expression

Sometimes, expression of a gene was observed throughout different tissues of a plant in WEB. In such cases, the validated phenotype of plants after knock out of the target gene in literature can be considered for reference. The abnormal cytokinin response1 repressor1(*TaARE1*) gene’s loss-of-function mutations in rice resulted in delayed senescence, enhanced nitrogen-use efficiency, and increased grain yield under N-limiting conditions (Wang et al., 2018; Zhang J. et al., 2021). The gene expression studies revealed that the *TaARE1* expression is not tissue specific and is expressed in different tissues in varied amounts. A couple of studies showed that the editing of *TaARE1* did not produce any undesired phenotypic effect in wheat and rice mutants (Wang et al., 2018; Zhang J. et al., 2021). Thus, it can be considered a potential candidate gene for SDN1 gene editing in wheat.

#### 3.2.3 Checking the conserved region of gene across sub-genomes

Based on the available reports, there are two different types of genes for SDN1 editing. The first category includes edits that delete any part of the target gene to cause its knockout; also, the target genes can have differences in size (kb) and the number of exons across sub-genomes. The second category includes edits that delete only a specific domain in the target gene to cause its knock out. Thus, identifying the unique protein-coding consensus sequence conserved across three wheat genomes is challenging for designing efficient gRNA for a target gene.

##### 3.2.3.1 Deleting any part of the target gene causes its knockout

**FIGURE 3:**
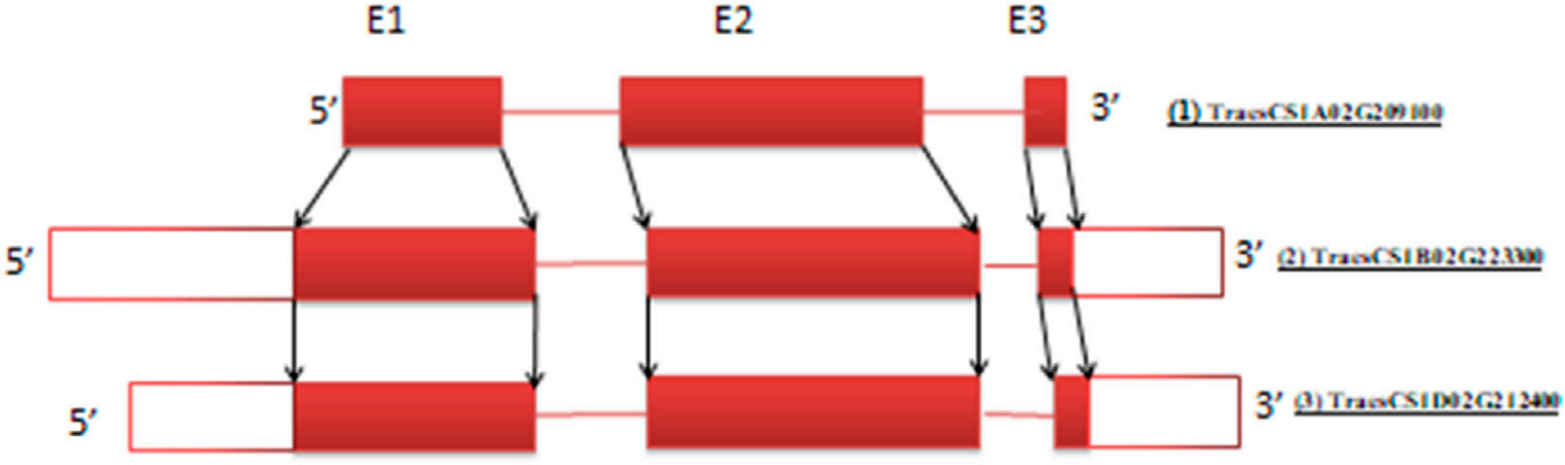
The *TaProDH* gene’s three exons display a high degree of conserved regions, as indicated by arrows across three sub-genomes. Gene IDs: **(1)** TraesCS1A02G209100 (1.64 kb), **(2)** TraesCS1B02G223300 (2.79 kb), and **(3)** TraesCS1D02G212400 (2.63 kb) (not to scale) (Solid red blocks represent exons while white blocks represent the untranslated region (UTR) of gene at the 5′ and 3′ ends.).

This is explained by taking the proline dehydrogenase (*ProDH*) gene, which negatively regulates thermo-tolerance in rice (Guo et al., 2020), as an example. This gene in wheat was found to have the same number of exons in all three genomes, and its cDNA sequence in three homeologs is highly conserved (>95%) (Table 2). Based on similarity among corresponding exons of homeologs, the conserved exons sharing similarity are shown in Figure 3. Usually, the number of exons in a gene and their size is constant across sub-genomes; however, variations in size (like the *TaProDH* gene) and the number of exons do exist in a few genes like *TaITPK1*. In such cases, consensus sequences across three sub-genomes can be identified by multiple sequence analysis, preferably in the initial exons of the genes for gRNA designing for knocking out of the gene.

**TABLE 2:** Percentage similarity of complementary DNA (cDNA) protein-coding sequence and coding sequence (CDS) of exons 1, 2, and 3 among the *ProDH* gene’s three homeologs in wheat.

Gene ID	cDNA protein-coding sequence	CDS protein-coding sequence	Exon 1	Exon 2	Exon 3
TraesCS1A02G209100	95.23	95.23	81.36	97.74	100
TraesCS1B02G223300	100	100	100	100	100
TraesCS1D02G212400	94.55	98.34	95.12	98.53	82.99

##### 3.2.3.2 Deleting a specific domain in the target gene causes its knock out

**FIGURE 4:**
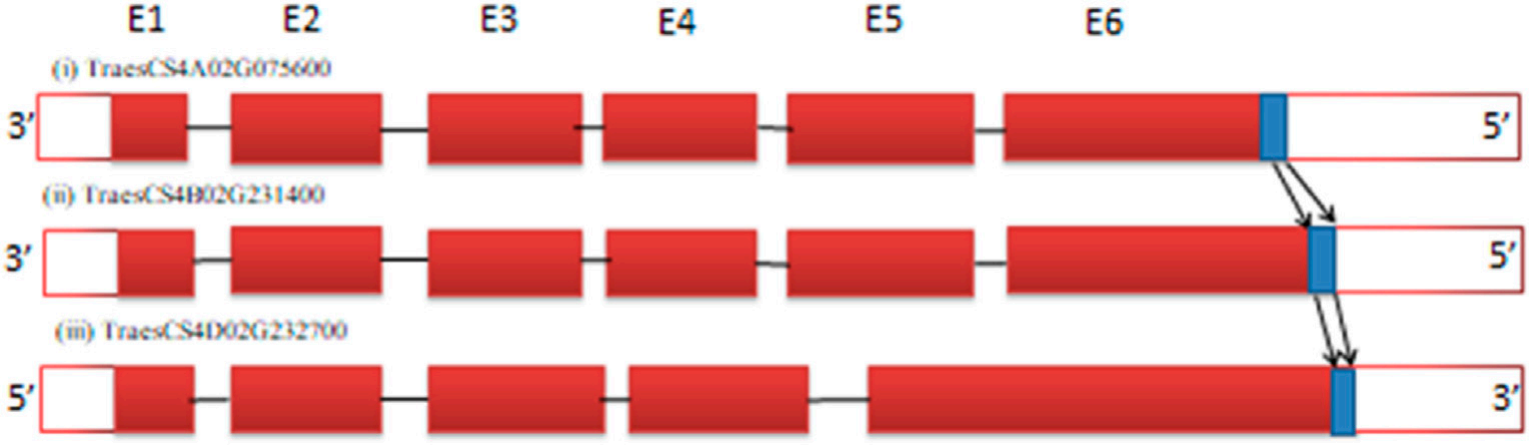
Location of the calcium modulin binding domain (CaMBD) (marked in blue) in the last exon of each homeolog of glutamate decarboxylase (*GAD3*) gene of wheat. Gene IDs: **(1)** TraesCS4A02G075600, **(2)** TraesCS4B02G231400, and **(3)** TraesCS4D02G232700 (not to scale) (solid red blocks represent exons while white blocks represent the untranslated region (UTR) of gene at the 5′ and 3′ ends).

Sometimes, only a specific domain of the target gene of only a few nucleotides (Nonaka et al., 2017; Lee et al., 2018; Akama et al., 2020) must be deleted to knock out gene function. This can be best explained with the calcium modulin binding domain (CaMBD) of the glutamate decarboxylase (*GAD3*) gene (Figure 4). The *GAD3* gene, which is responsible for increasing γ-aminobutyric acid (GABA) content, contains an auto-inhibitory domain. Knocking down the C-terminus, which includes this auto-inhibitory CaMBD domain, allows the enzyme to become constitutively active, thereby increasing GABA content in crops like rice and tomato (Nonaka et al., 2017; Lee et al., 2018; Akama et al., 2020). In wheat, the CaMBD domain of *GAD3* is made up of 87 nucleotides that encode 29 amino acids (Figure 4). This domain is highly conserved, with a 97.7% nucleotide sequence similarity and an identical peptide sequence. Due to the small size of this target sequence (87 nucleotides), there are only six protospacer adjacent motif (PAM) sites available for designing gRNAs. Similar auto-inhibitory domains can be found in other genes within the wheat genome, and for these genes, gRNA should be specifically designed to target the domain for effective gene knockout.

### 3.3 gRNA designing

Detailed steps for designing the most suitable gRNA are explained by considering the *TaARE1* gene as an example. Before designing gRNA for *TaARE1*, the gene location on chromosomes was searched from literature (or it could be identified using the KnetMiner *Triticum aestivum* bioinformatics tool). *TaARE1* is located on chromosomes 7A, 7B, and 7D (Wang et al., 2018; Zhang J. et al., 2021). The sequence of the gene was obtained from the EnsemblPlants database, consisting of seven exons and six introns. While checking for similarity and designing gRNA, only exons, that is, the coding portions of the gene, were considered. All the homeologs of the *TaARE1* gene’s protein-coding regions and corresponding exons displayed high similarity (exonic similarity ranged from 97.96% to 100%), thereby facilitating designing common gRNA targeting the three genomes at once. The gene IDs identified for the *TaARE1* gene are TraesCS7A02G286400, TraesCS7B02G196800, and TraesCS7D02G283700 for the three sub-genomes.

**FIGURE 5:**
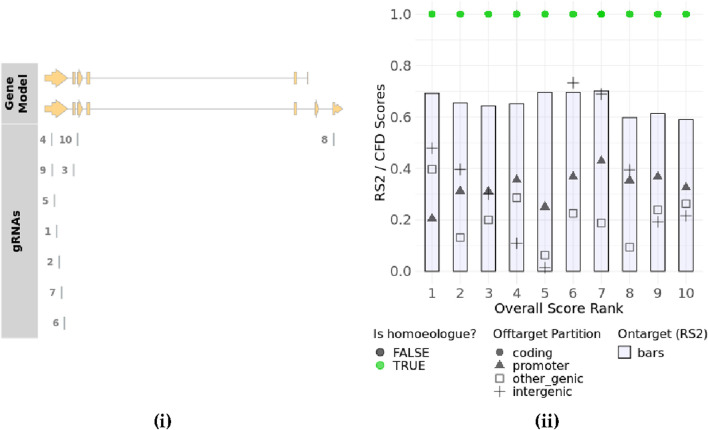
Gene plot showing the location of designed top 10 gRNAs in the corresponding exons of two sub-genomes for the *TaARE1* gene (taken from https://crispr.bioinfo.nrc.ca/WheatCrispr/) **(i)** gRNAs plot (Overall score rank and rs2/CFD scores) (taken from https://crispr.bioinfo.nrc.ca/WheatCrispr/) **(ii)**.

WheatCRISPR software (https://crispr.bioinfo.nrc.ca/WheatCrispr/) was used to design the gRNA. It displayed a list of the top 10 gRNAs based on overall score rank after giving the input gene name as *ARE1* or an identified gene ID like TraesCS7D02G283700 along with targeting homeologs (Figure 5).

In this software, gRNAs are scored according to the predicted on-target activity and off-target potential using pre-determined models (Doench et al., 2016). The software provides rule set 2 (rs2) scores that measure the predicted cutting efficiency of the gRNA. It is a machine-learning-based score that predicts the cutting efficiency of a gRNA at the intended target site determined by the position of nucleotides within the guide sequence or the presence of specific motifs that enhance or reduce activity. A higher rs2 score indicates better on-target activity. In addition, it provides cutting frequency determination (CFD) scores for coding, promoter, inter-genic, and genic regions (Cram et al., 2019) (Table 3). The CFD score predicts the likelihood of Cas9 cutting at off-target sites based on sequence mismatches by considering mismatch tolerance between the guide RNA and potential off-target sites. Some mismatches are more tolerated than others, depending on their position in the gRNA. Higher scores indicate higher off-target cutting risk. CFD scores for different types of genomic regions, like a coding region, a promoter region, and an intergenic region, predict the impact of off-target mutations within exons (which may cause functional changes in protein sequences), off-target effects in regulatory sequences (which can alter gene expression), or off-target effects in non-coding DNA (which may have minimal functional consequences), respectively. The CFD score for a genic region considers all regions within genes, including exons, introns, and UTRs. CFD scores by region help in choosing gRNAs with the intended location.

**TABLE 3:** First ten gRNA designed for the *TaARE1* gene.

S.No	Rule set (rs2) score	Coding region cutting frequency determination (CFD) score	Promoter region CFD score	Other genic region CFD score	Intergenic CFD score	Gene ID/genomic location of potential off-target hit having PAM 5′-NGG-3′ and/or for k < 3	Percent GC content in gRNA	Exon number of gene in which gRNA is located
1	0.69	0.22	0.20	0.40	0.48	TraesCS3D02G317400	60	1
						TraesCS3B02G353000		
						7D: 404067180-404067196		
2	0.66	0.29	0.31	0.13	0.40	5A: 542540704-542540720	45	1
3	0.64	0.26	0.31	0.20	0.30	3B: 37075187 to 37075204	45	2
						6B: 629944421 to 629944438		
						1A: 447098576 to 447098592		
						Un 275940833 to 275940849 7A: 299827040 to 299827056		
						3B: 72737304 to 72737320		
						3A: 100244209 to 100244225		
						4B: 595815245 to 595815261		
4	0.65	0.23	0.35	0.29	0.11	0	40	1
5	0.70	0.51	0.25	0.063	0.014	0	60	1
6	0.70	0.36	0.37	0.23	0.73	5A: 279741731 to 279741748	50	1
						6D: 459806940 to 459806956		
						7B: 77936349 to 77936369		
7	0.70	0.33	0.35	0.094	0.39	0	45	1
8	0.60	0.27	0.37	0.24	0.19	0	55	7
9	0.61	0.33	0.33	0.26	0.22	0	40	1
10	0.59	0.38	0.36	0.053	0.0	0	40	3

In the top 10 gRNAs, scores ranged from 0.59 to 0.70, with higher-ranked gRNAs showing better on-target activity. Ideally, three hits should occur in specific sub-genome loci that are targeted, while others should be minimized. To assess off-target risks, gRNAs (20 nucleotides) plus the PAM sequence were analyzed using BLAST software against the wheat genome. Off-target hits were identified based on mismatch patterns, with at least two mismatches in the PAM-proximal region needed to disregard hits as off-targets. Mismatches at the 5′ end were more tolerable unless the rest of the sequence matched the 3′ end. No perfect off-target matches were found. Only one gRNA had a potential off-target with two mismatches at location 7B: 77936349 to 77936369. Other gRNAs showed a minimum of four mismatches. The percent Guanine-cytosine (GC) content for gRNAs ranged from 45% to 60%.

### 3.4 gRNA analysis

The intra-base pairing in the gRNA may interfere with its target recognition (Liang et al., 2016). The analysis indicated that 35% of gRNAs contained at least one internal base pairing. Hence, gRNA forming no internal base pairing should ideally be for efficient editing. This can be estimated by predicting the secondary structure of gRNA, the change in Gibbs free energy, and by calculating the propensity of gRNA to remain single stranded based on the potential secondary structure. This will effectively help us to screen the most effective gRNA from those that would make the editing process non-specific.

#### 3.4.1 Analyzing gRNA for secondary structure formation

The secondary structure formation of gRNA was analyzed using Mfold software (http://www.bioinfo.rpi.edu/applications/mfold) under the following conditions: 37°C, Na^+^ = 1.00 M, and Mg^2+^ = 0.0 M (Zuker, 2003). The standard Gibbs free energy change, ΔG (delta G), indicates the thermodynamic favorability of a physical or chemical process, such as the folding of gRNA into a secondary structure. When ΔG < 0, the process is thermodynamically favored. The predicted values of ΔG indicate whether gRNA will form a secondary structure spontaneously (negative ΔG) or not (positive ΔG) from Equation 1.

ΔG=ΔH – TΔS (1)

(where ΔH is enthalpy, T is the temperature in Kelvin, and ΔS is entropy).

**FIGURE 6:**
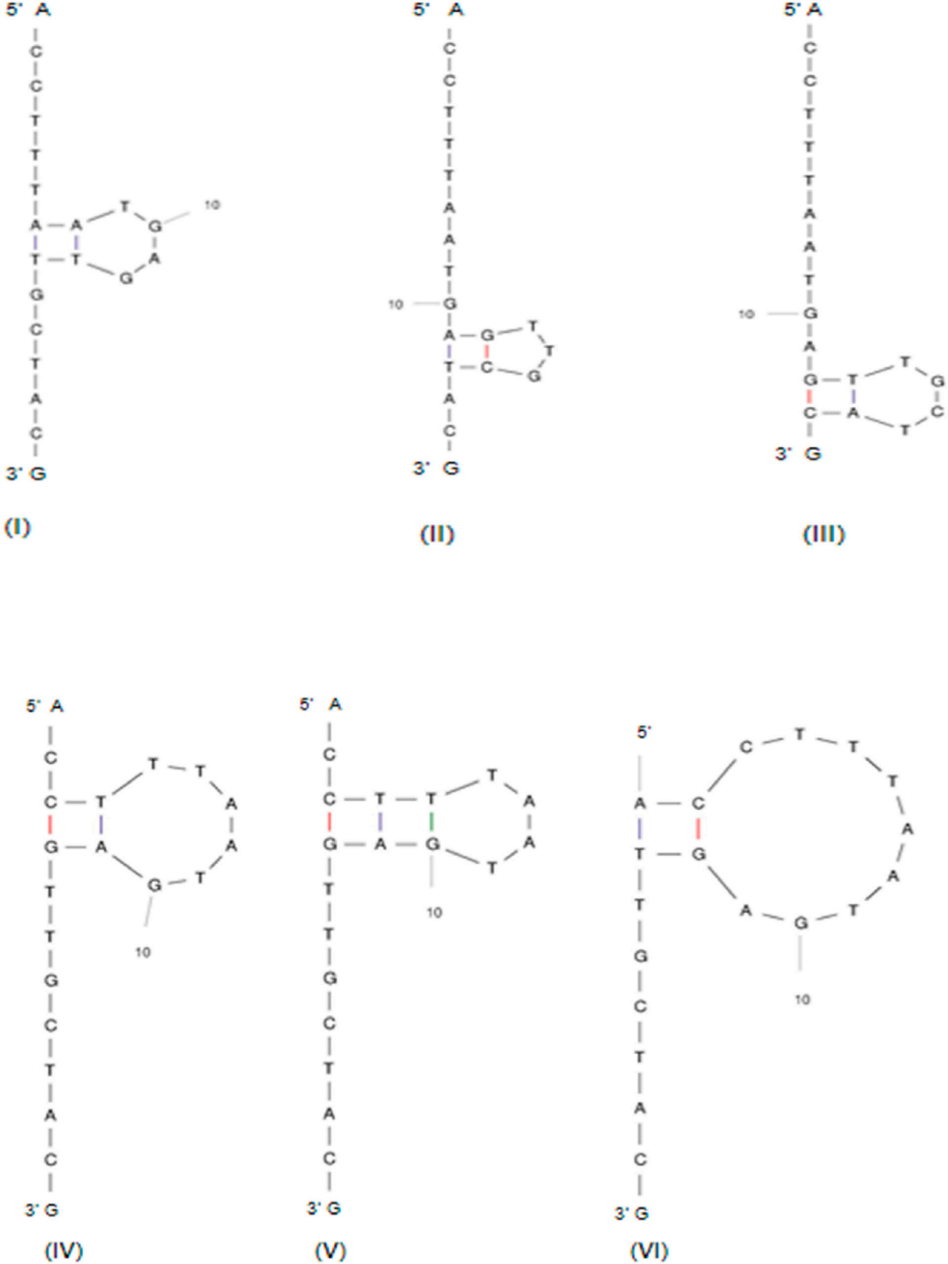
Six possible secondary structures of *TaARE1* gRNA 4 at 37°C with **(I)** ΔG = +0.84 kcal/mol, **(II)** ΔG = +1.17 kcal/mol, **(III)** ΔG = +1.17 kcal/mol, **(IV)** ΔG = +1.25 kcal/mol, **(V)** ΔG = +1.49 kcal/mol, and **(VI)** ΔG = +1.82 kcal/mol (http://www.bioinfo.rpi.edu/applications/mfold).

This is explained by taking the gRNAs of the gene *TaARE1* (Table 3). The first ten designed gRNAs were checked for their potential secondary structure formation due to intra-base pairing (Table 4). The ΔG of the fourth gRNA of *TaARE1* varied from +0.84 to +1.82 kcal/mol (Figure 6), indicating a higher likelihood that the gRNA would stay in a linear state rather than folding and forming a secondary structure. This gRNA will not form secondary structures due to positive ΔG values, which is preferred for effective gRNA. The other gRNAs with negative ΔG values will spontaneously fold to form secondary structures, and hence, they are undesirable. In such cases, alternative gRNA should be explored. *TaITPK1* gene’s first 10 gRNAs are also included as another example of secondary structure formation (Table 4).

**TABLE 4:** Potential secondary structures, free Gibbs energy, and number of gRNA bases having a single-stranded propensity for the top 10 gRNAs of *TaARE1* and *TaITPK1*.

S.No.	gRNA sequence (5′–3′)	Number of potential secondary structures (as per Mfold software)	Free Gibbs energy range of gRNA (kcal/mol) (as per Mfold software)	Number of bases with propensity to remain single-stranded in gRNA (as per SS-count prediction)
*TaARE1*				
1	GAG​AAC​CAC​GCC​TTC​CAC​CA	4	+0.91 to +1.68	20
2	TGT​TAG​CAA​CGA​AGA​CCT​GT	7	+0.44 to +1.43	20
3	ACT​AGA​AGA​TCC​AAC​ACC​CA	2	0.39 to +1.36	20
4	ACC​TTT​AAT​GAG​TTG​CTA​CG	6	+0.84 to +1.82	20
5	TGT​TCG​ATG​TCG​GTC​CCC​AG	2	−0.69 to +0.16	16
6	TGA​TCC​GAC​GGA​GAT​AGT​GA	3	−0.10 to +0.79	20
7	TCC​AAG​ATG​TTT​CCC​ACA​CT	1	−0.34	—
8	TGC​TAC​GTG​GTC​AGC​TCT​AG	1	−0.75	—
9	CAA​TAA​CAG​GTA​TGT​CGA​GA	1	−0.64	—
10	AAA​TGC​AGA​AGG​GCG​GTC​AT	4	+0.14 to +1.12	20
*TaITPK1*				
1	GAA​GGA​GCT​ACT​CAA​TGT​TG	2	−0.46 and +0.01	19
2	GTC​AAA​AGA​AAG​AAT​ACG​CA	5	1.80 to 2.65	20
3	AAT​ATC​TAA​ATC​TGG​AGT​AT	7	1.27 to 2.23	20
4	CAT​GGG​AGG​TGG​TGC​TGA​TA	3	0.65 to 1.32	20
5	GGA​TGA​TCA​TGA​CAT​CGA​AG	1	−0.90	—
6	TTG​ATC​GAG​TCT​GTC​AAG​CA	7	0.09 to 0.98	20
7	ATG​TCA​TGA​GCC​AAC​ATC​TG	2	−0.11 and +0.11	17
8	ACA​GCT​TGG​CAA​GGT​ACT​GC	7	−0.74 to +0.11	20
9	CAA​TTT​CTA​CAC​TAA​GAG​CG	4	+0.62 to +1.28	20
10	GTG​TCT​TGA​AGA​TCT​GAG​GA	5	+0.77 to +1.40	20

The ss-count parameter from Mfold software (Zuker, 2003) predicts how likely it is for a gRNA to stay single stranded or to form secondary structures. If only one structure is possible, the ss-count is not calculated to avoid bias. Instead, an ss-count is done for all 20 base pairs of the gRNA to see how likely it is to form a secondary structure. A higher number of bases with a propensity to be single stranded directly translates to a gRNA that is more likely to remain single stranded and not form an undesirable secondary structure; it also depends on the composition of bases of the gRNA, as the length of gRNAs almost remains constant (∼20 bases).

**FIGURE 7:**
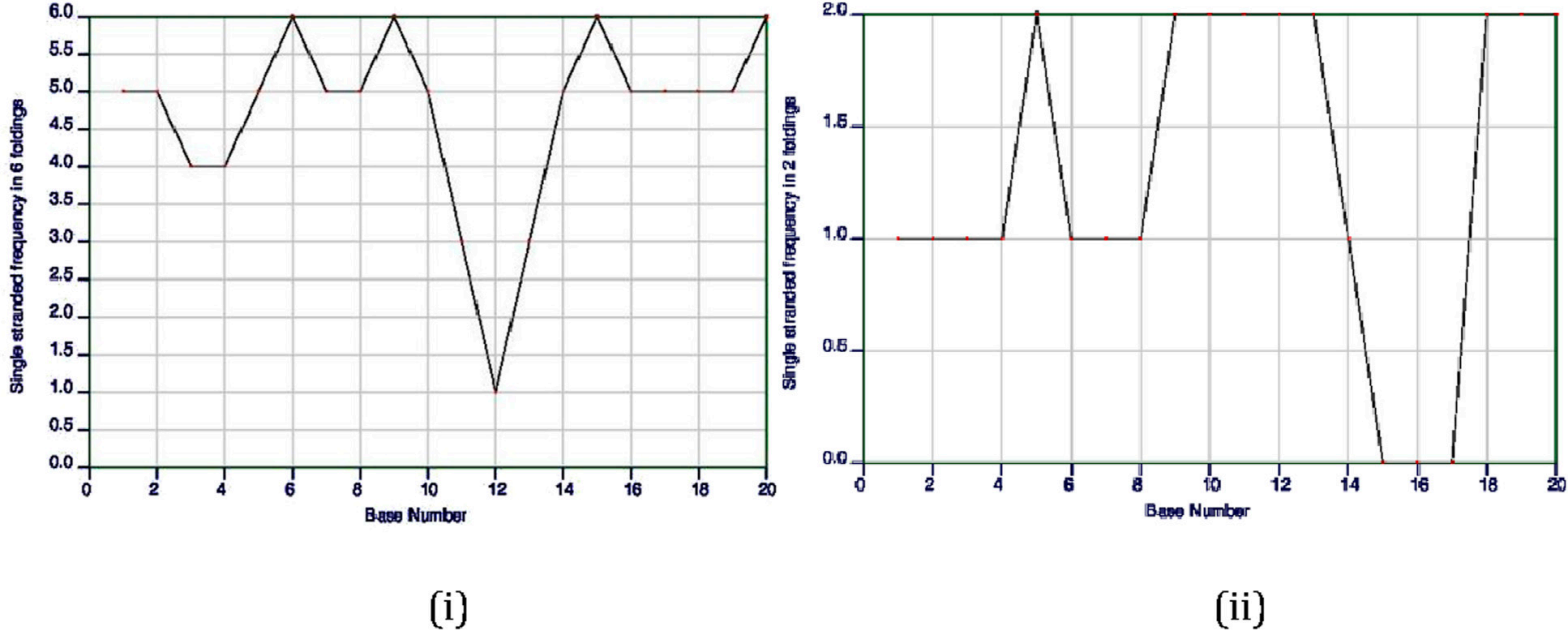
Single-stranded (ss)-count plot of *TaARE1’s* 4th gRNA based on six possible secondary structures **(i)** and *TaITPK’s* 7th gRNA based on two possible secondary structures (http://www.bioinfo.rpi.edu/applications/mfold) **(ii)**.

Of the first 10 gRNA candidates for *TaARE1*, gRNAs 1, 2, 3, 4, and 6 showed no signs of forming double strands, meaning they likely will not form secondary structures. On the other hand, gRNA 5 showed that 16 of its 20 bases were likely to stay single stranded, but further tests revealed it could still form a secondary structure due to some bases not remaining single stranded. Figure 7 shows the ss-count plot for gRNA 4 of *TaARE1* and gRNA 7 of *TaITPK*.

#### 3.4.2 Checking vector backbone homology with the gRNA sequence

The designed gRNA should be checked for sequence homology in the desired vector backbone as it may cause multiple amplifications after cloning. Due to the co-amplification of gRNA along with complementary sequences in the vector, multiple amplifications will be seen after cloning of the gRNA into the vector. Hence, the designed gRNA should be checked for vector complementarity using SnapGene Viewer (GSL Biotech, 2020), and the gRNA with no complementarity/sequence homology should be preferably selected for further cloning.

#### 3.4.3 Confirmation of the absence of a transcription terminator sequence in the gRNA

The expression of gRNA into the plant system is commonly driven by small nuclear RNA gene promoters (U3 or U6). The transcription of gRNA is done through RNA polymerase III (Jiang et al., 2013). The designed gRNA should not contain any transcription termination sequence like a poly-T (thymine) tail at the 3′ end (Jiang et al., 2013) for the U3 or U6 promoter (Richard and Manley, 2009).

This study outlines a novel methodology for designing effective guide RNAs (gRNAs) for SDN1-mediated CRISPR/Cas9 genome editing in wheat, specifically addressing the challenges posed by its complex polyploid genome. The methodology involves three key phases: Target gene identification and verification, gRNA design, and gRNA analysis. We focused on selecting target genes that are negative regulators of important agronomic traits, such as drought tolerance and yield, ensuring that gene knockouts would lead to desirable phenotypic changes. Using WheatCRISPR software, we designed gRNAs with high on-target activity, low off-target potential, and optimal cutting efficiency.

To further refine gRNA selection, we conducted a thorough *in silico* analysis, including secondary structure prediction using Mfold software. This step was crucial in identifying gRNAs that are likely to remain linear and functional, thereby reducing the risk of forming secondary structures that could impede genome editing. Our results demonstrated that the gRNAs designed through this methodology showed minimal off-target effects and high specificity for their target sequences, particularly in the context of the wheat genome’s repetitive DNA and polyploid nature. The successful application of this methodology has significant implications for wheat breeding, offering a robust framework for improving key traits through precise genome editing.

## 4 Conclusion

The development and application of novel and comprehensive techniques for designing precise guide RNAs (gRNAs) mark a significant advancement in SDN1-mediated genome editing for wheat. These optimized methodologies enhance the accuracy, efficiency, and specificity of targeted edits, addressing key challenges inherent to plant genome editing. By refining gRNA design, we enable precise modifications with minimal off-target effects, paving the way for targeted improvements in wheat, including stress resistance, yield optimization, and quality enhancement. This work not only contributes to a better understanding of the SDN1 mechanism but also provides a valuable toolkit for researchers aiming to enhance genetic improvements in wheat and other crops. These advancements hold promise for accelerating the development of improved wheat varieties, thus contributing to global food security and sustainable agriculture. More pangenome sequence information in diverse wheat cultivars and field testing of edited plants are needed to validate these findings and optimize gRNA design tools for broader application in crop improvement.

## Data Availability

The original contributions presented in the study are included in the article/supplementary material; further inquiries can be directed to the corresponding authors.
